# Association between cerebral cannabinoid 1 receptor availability and body mass index in patients with food intake disorders and healthy subjects: a [^18^F]MK-9470 PET study

**DOI:** 10.1038/tp.2016.118

**Published:** 2016-07-12

**Authors:** J Ceccarini, N Weltens, H G Ly, J Tack, L Van Oudenhove, K Van Laere

**Affiliations:** 1 Division of Nuclear Medicine and Molecular Imaging, Department of Imaging and Pathology, University Hospitals Leuven, KU Leuven, Leuven, Belgium; 2 Department of Clinical and Experimental Medicine, Translational Research Center for Gastrointestinal Disorders, KU Leuven, Leuven, Belgium; 3 Department of Gastroenterology, University Hospitals Leuven, Leuven, Belgium; 4 Liaison Psychiatry, University Psychiatric Center Campus Gasthuisberg, University Hospitals Leuven, Leuven, Belgium; 5 Leuven Institute for Neurobiology and Disease, KU Leuven, Leuven, Belgium

## Abstract

Although of great public health relevance, the mechanisms underlying disordered eating behavior and body weight regulation remain insufficiently understood. Compelling preclinical evidence corroborates a critical role of the endocannabinoid system (ECS) in the central regulation of appetite and food intake. However, *in vivo* human evidence on ECS functioning in brain circuits involved in food intake regulation as well as its relationship with body weight is lacking, both in health and disease. Here, we measured cannabinoid 1 receptor (CB_1_R) availability using positron emission tomography (PET) with [^18^F]MK-9470 in 54 patients with food intake disorders (FID) covering a wide body mass index (BMI) range (anorexia nervosa, bulimia nervosa, functional dyspepsia with weight loss and obesity; BMI range=12.5–40.6 kg/m^2^) and 26 age-, gender- and average BMI-matched healthy subjects (BMI range=18.5–26.6 kg/m^2^). The association between regional CB_1_R availability and BMI was assessed within predefined homeostatic and reward-related regions of interest using voxel-based linear regression analyses. CB_1_R availability was inversely associated with BMI in homeostatic brain regions such as the hypothalamus and brainstem areas in both patients with FID and healthy subjects. However, in FID patients, CB_1_R availability was also negatively correlated with BMI throughout the mesolimbic reward system (midbrain, striatum, insula, amygdala and orbitofrontal cortex), which constitutes the key circuit implicated in processing appetitive motivation and hedonic value of perceived food rewards. Our results indicate that the cerebral homeostatic CB_1_R system is inextricably linked to BMI, with additional involvement of reward areas under conditions of disordered body weight.

## Introduction

Dysregulations of appetite, eating behavior and body weight are hallmark symptoms of a wide range of chronic and disabling illnesses that can collectively be referred to as food intake disorders (FID).^1^ In this sense, FID include obesity (OB) and eating disorders such as anorexia nervosa (AN) and bulimia nervosa (BN).^2, 3^ The core behavioral features of these disorders include either food avoidance or excessive food intake,^2^ which may be accompanied by compensatory behavior (that is, fasting, excessive physical exercise, vomiting and/or laxative/diuretic use) intended to control weight (especially in AN and BN). This, in turn, might underlie abnormalities in body mass index (BMI), ranging from extreme underweight to morbid OB. Moreover, functional dyspepsia (FD), a prevalent functional gastrointestinal disorder characterized by meal-induced epigastric symptoms, is often accompanied by disturbed appetite and food intake as well as unintentional weight loss.^4^ Together, these disorders represent major global health problems that put an enormous demand on health-care services, not at least because of their high medical comorbidity.

During the last decade, the endocannabinoid system (ECS) emerged as one of the most important neuromodulatory systems involved in both the central and peripheral regulation of food intake and body weight.^5, 6^ The cerebral type 1 cannabinoid receptor (CB_1_R) is the most abundant G-protein-coupled receptor in the central nervous system, where it resides predominantly at presynaptic nerve terminals to directly or indirectly modulate glutamatergic and GABAergic neurotransmission.^7^ It is now well accepted that stimulation of cannabinoid receptors by synthetic or plant-derived ligands such as Δ^9^-tetrahydrocannabinol (Δ^9^-THC) increases appetite and food intake in both humans and laboratory animals, especially toward foods with high palatability.^8, 9^ Conversely, pharmacological blockade of CB_1_R reduces hunger, food intake and body weight of patients with OB.^10, 11^ Animal experiments indicate that these effects result in large part from targeting CB_1_R in several interconnected brain circuits linking homeostatic centers in the brainstem and hypothalamus with the mesolimbic reward system that encompasses ventral tegmental area, striatum, amygdala, hippocampus and orbitofrontal cortex.^5, 12^ Together, these neural structures represent the major integration centers for the regulation of appetite and food intake, where the ECS is believed to modulate energy homeostasis, reward sensitivity and motivated behavior.^13, 14^ Specifically, it appears that endocannabinoids not only regulate the expression and release of hypothalamic orexigenic and anorexigenic signals, but also modulate activity in mesolimbic dopaminergic incentive pathways and opioidergic hedonic circuits, hence facilitating appetitive motivation as well as the pleasure of food during ingestion.^15, 16, 17, 18^

Given the direct involvement of the ECS in the central neurocircuitry mediating energy homeostasis and food reward, it is not surprising that increasing evidence points toward disturbed endocannabinoid signaling in FID. Several reports indicate differences in plasma and/or tissue endocannabinoid levels as well as altered central CB_1_R availability in both obese and anorectic conditions.^9, 19, 20, 21, 22, 23, 24^ Moreover, specific genetic variants of several ECS components have been associated with AN, BN and OB.^25, 26, 27, 28, 29^ However, despite mounting evidence supporting disturbed ECS signaling in several separate pathological eating- and weight-related conditions, so far there are no *in vivo* human studies linking endocannabinoid function in the key food intake-related brain areas to body weight along the BMI spectrum.

In this study, we used positron emission tomography (PET) imaging with the selective CB_1_R radioligand [^18^F]MK-9470^30^ to investigate for the first time whether *in vivo* cerebral CB_1_R availability in the key homeostatic and reward-related brain areas is associated with BMI in patients with FID covering a wide BMI range (AN, BN, FD with severe weight loss and OB), and in healthy subjects within the normal BMI range. As a second objective, conjunction and interaction analyses were performed to investigate whether potential CB_1_R–BMI associations would differ between both groups.

## Materials and methods

The study was approved by the local ethics committee of the University Hospital and KU Leuven and was performed according to the latest version of the World Medical Association Declaration of Helsinki. All subjects provided written informed consent after receiving a full explanation of the study procedures.

### Subjects

A total of 54 FID patients with large BMI range (mean±s.d. BMI 22.6±8.0 kg/m^2^, BMI range 12.5–40.6 kg/m^2^, mean±s.d. age 29.3±12.6 years) and 26 healthy subjects of normal weight (mean±s.d. BMI 22.3±2.4 kg/m^2^, BMI range 18.5–26.6 kg/m^2^, mean±s.d. age 34.6±15.3 years) participated in the study. FID patients included those with AN (*n*=14), BN (*n*=16), FD with severe weight loss due to loss of appetite (*n*=12) and OB (*n*=12). Demographic data for all subjects are summarized in Table 1. There were no significant differences (Table 2) between the two groups for age (*P*=0.10), sex (*P*>0.99), average BMI (*P*=0.87) and injected radioligand dose (*P*=0.15).

The patient sample of the present study does partially overlap with two recent studies by our group.^23, 31^ However, the hypothesis tested in this present study is completely novel and has not been reported elsewhere. Full details on AN, BN and FD patient selection are available in the Supplementary Material. OB patients were recruited by their primary care physicians and had a BMI⩾30 kg/m^2^. A neuropsychological assessment was performed using several questionnaires, and they were screened for exclusion criteria such as comorbid BN and binge-eating disorder using the Structured Clinical Interview for DSM-IV Axis I psychiatric disorders (SCID).^32^ Furthermore, their body weight had been stable for at least three consecutive months before the study, and they had not undergone any behavioral, therapeutic or surgical treatment aiming at or leading to weight loss for at least three consecutive months.

All FID patients were screened for absence of other neuropsychiatric or medical conditions, and were free of any (psychotropic or other) medications and/or (recreational) drugs, and any substance abuse or dependence that might influence CB_1_R levels. Absence of drug use was confirmed by blood and urine testing on the day of scanning, including general screening and toxicology tests for benzodiazepines, neuroleptics, opioids, cocaine, metabolites, amphetamine and cannabinoids.

Healthy control subjects were selected randomly from previous CB_1_R PET studies based on the average BMI of the FID group^33, 34, 35^ to obtain a sample that was matched to the patient cohort for age, gender and average BMI (Table 1). All controls were free of diagnosable psychopathology according to DSM-IV criteria, and inclusion and exclusion criteria were as described previously.^33^

### Image acquisition

CB_1_R imaging was performed using the radioligand [^18^F]MK-9470, which is an inverse agonist with high affinity and specificity for the CB_1_R.^30^ The [^18^F]MK-9470 precursor was obtained from Merck Research Laboratories (MRL, West Point, PA, USA) and labeling was performed on-site using 2-[^18^F]fluoroethylbromide. Tracer synthesis, characteristics and administration procedure were described previously.^30^ The final product was obtained after high-performance liquid chromatography separation and had a radiochemical purity>95%.

All subjects fasted for at least 4 h before their PET session. To minimize intrascan head movement, subjects were positioned in the scanner gantry with the head placed in a vacuum cushion and the body fixed before start of the dynamic emission scan. Each subject received on average 291.1±47.4 MBq of [^18^F]MK-9470 in slow bolus intravenous injection, under standardized injection circumstances (mean±s.d. 285.8±51.6 MBq for FID patients, 302.0±35.7 MBq for control subjects). CB_1_R images were acquired in a three-dimensional mode using a ECAT EXACT HR+ PET camera (Siemens, Erlangen, Germany) for all AN, BN and FD patients as well as 14 controls, and a HiRez Biograph 16 PET/CT camera (Siemens, Knoxville, TN, USA) for all OB patients and the remaining 12 controls.

PET acquisition on the HR+ PET camera started 90 min post injection with 30-min scanning (six frames of 5 min), while the scanning protocol on the HiRez PET/CT camera consisted of a 60-min acquisition starting 120 min post injection (six frames of 10 min). These small differences in acquisition conditions pose no problem for further analyses, as [^18^F]MK-9470 brain kinetics reach a plateau between 90 and 120 min post injection and remain relatively stable up to 460 min.^36^ Moreover, to exclude potential intercamera differences in CB_1_R assessment, we performed all analyses with camera as additional covariate of no interest.

HR+ PET images were reconstructed using the three-dimensional filtered back-projection algorithm including scatter and measured attenuation correction (^68^Ge source). For the PET data acquired on the HiRez PET/CT camera, a low-dose (80 kV tube potential, 11 mAs) CT scan without contrast agent was performed at the beginning of each PET scan for attenuation correction. Images were reconstructed using a three-dimensional OSEM (ordered-subset expectation maximization) iterative reconstruction with five iterations and eight subsets including scatter and attenuation correction. The resulting transverse and axial spatial resolution for both systems was ~4 mm.

In addition, all subjects underwent a structural magnetic resonance imaging (MRI) scan, both T1-weighted Magnetization Prepared Rapid Acquisition Gradient Echo and T2-weighted, to exclude structural brain abnormalities and to anatomically co-register with the PET images. MRI data were acquired on a 1.5-Tesla Vision Scanner (Siemens).

### Image processing

CB_1_R availability was quantified using the modified standardized uptake value (mSUV) as index, a previously validated and non-invasive simplified quantification method that does not require invasive blood sampling.^36^ mSUV normalizes the calibrated radioactivity concentration at each voxel with injected radioactivity dose and subject’s weight: mSUV=(activity concentration (KBq/cc) × (subject’s body weight (kg)+70)/2)/injected dose (MBq).^37^ In this way, body weight was additionally normalized to a reference weight (that is, average body weight of an adult person (70 kg)) to account for the large weight difference between the groups. Hence, the systematical underweight of AN patients would imply an underestimation of CB_1_R availability, whereas the overweight of OB subjects would result in an overestimation.

Moreover, mSUV gives a reliable estimate of the total distribution volume (*V*_T_) of [^18^F]MK-9470, as determined by full kinetic modeling in humans^36^ and healthy rats^24^ under the condition that group differences in peripheral tracer metabolism and tissue distribution can be excluded. The absence of such group differences in metabolite-corrected input function and peripheral tracer metabolism has been demonstrated in subsets of these patients^23^ as well as the activity-based rat model of AN.^24^ However, in order to assess the validity of mSUV in this study, the fractional uptake ratio, which is an index strongly proportional to the total *V*_T_ of [^18^F]MK-9470, was calculated as the ratio of tracer concentration in tissue at the end of the scan to the integral of metabolite-corrected plasma activity from time of injection to the end of the scan.^36^ To obtain the metabolite-corrected input curve, [^18^F]MK-9470 plasma concentration and [^18^F]MK-9470 percentage fractions were measured for a subgroup of FID patients (*n*=10) and control subjects (*n*=10) with venous sampling between 0 and 120 min post injection. This procedure and [^18^F]MK-9470 metabolite determination were performed as described earlier.^36^ The direct relation between regional mSUV and fractional uptake ratio values in cortical and subcortical grey matter regions of interest (ROIs) showed a very strong correlation (*R*=0.99; Supplementary Figure 1), thereby excluding possible group differences in peripheral metabolism that could lead to bias in CB_1_R availability determination by the simplified quantification mSUV. This indicates that no significant bias in the mSUV versus fractional uptake ratio relationship was present between FID patients and controls, and mSUV can be used as reliable indicator of *V*_T_.

For each subject, correction for motion between PET frames was performed in SPM8 (Statistical Parametric Mapping, Wellcome Department of Cognitive Neuroscience, London, UK), running on Matlab 7.1 (MathWorks, Natick, MA, USA). The motion-corrected [^18^F]MK-9470 mSUV images were then co-registered to the corresponding subject’s MRI with a mutual information algorithm, and then spatially normalized to a specific CB_1_R template constructed in Montreal Neurological Institute space (2 × 2 × 2 mm) using nonlinear warping. Individual normalized PET images were masked within the brain 80% isocontour of the CB_1_R template and were then smoothed at a full-width half maximum of 10 mm.

### Data analysis

On the basis of the substantial amount of (pre)clinical evidence on the involvement of the ECS in the regulation of food intake and energy balance,^5, 6, 38, 39^ an anatomical mask consisting of 11 *a priori* defined key homeostatic (that is, hypothalamus, pons and medulla) and reward/hedonic (that is, midbrain, nucleus accumbens, caudate head, putamen, pallidum, orbitofrontal cortex, insula and amygdala) areas was created using atlases available in the WFU-PickAtlas toolbox in SPM8.^40^ The full list of predefined ROIs comprising the mask is shown in Supplementary Table 1.

The sample sizes used in this study (*n*=54 for FID, *n*=26 for healthy controls) provided 80% power to detect significant correlations of moderate effect sizes (0.3–0.5) in each group with an alpha of 0.05 (two-sided).

Voxel-based linear regression analyses within the above-mentioned mask of predefined ROIs were performed using SPM8 to assess the association between CB_1_R availability and BMI in both groups. Owing to the right-skewed distribution of the BMI data within the FID group, BMI was first transformed by a natural logarithm to reduce the influence of potential outliers. Log-transformed BMI (log BMI) was then entered as a covariate in an analysis with group (FID, controls) and camera (HR+, HiRez) as factors and modeled voxel-wise against the mSUV CB_1_R data. This allowed regression analysis in both groups separately, corrected for potential intercamera variability. Results were examined at a voxel-level threshold of *P*_height_<0.05 family-wise error (FWE) corrected (corresponding with *T*>4.02) and additional cluster-level threshold of *P*_FWE-corrected_<0.05.

To validate our group results, exploratory regression analyses in each FID subgroup (that is, AN, BN, FD and OB) were performed at an uncorrected threshold of *P*_height_<0.05 to look whether similar patterns could be identified within each FID subgroup at a lower threshold.

Furthermore, voxel-wise conjunction and interaction analyses for the log BMI-mSUV relationship in both groups were performed within the ROI mask, both voxel- and cluster-level thresholded at *P*_FWE-corrected_<0.05. Conjunction analysis allows determining whether there are brain areas within the mask with significant negative correlations across the two conjoined groups, whereas interaction analysis identifies potential areas where the negative correlation between mSUV and log BMI is significantly different in FID patients compared with healthy controls.

Finally, to further confirm and illustrate the voxel-based regression, first eigenvariates from a 5-mm-radius sphere centered on the local maximum of those brain areas showing significant relationships in the SPM analysis were extracted using the eigenvariate procedure implemented in SPM. Linear regression coefficients relating CB_1_R availability (represented by the first eigenvariates extracted from the parametric mSUV maps) to log BMI were then determined using SAS 9.3 (SAS Institute, Cary, NC, USA) for each group.

## Results

### Association between cerebral CB_1_R availability and BMI

#### Group analysis

**Figure 1:**
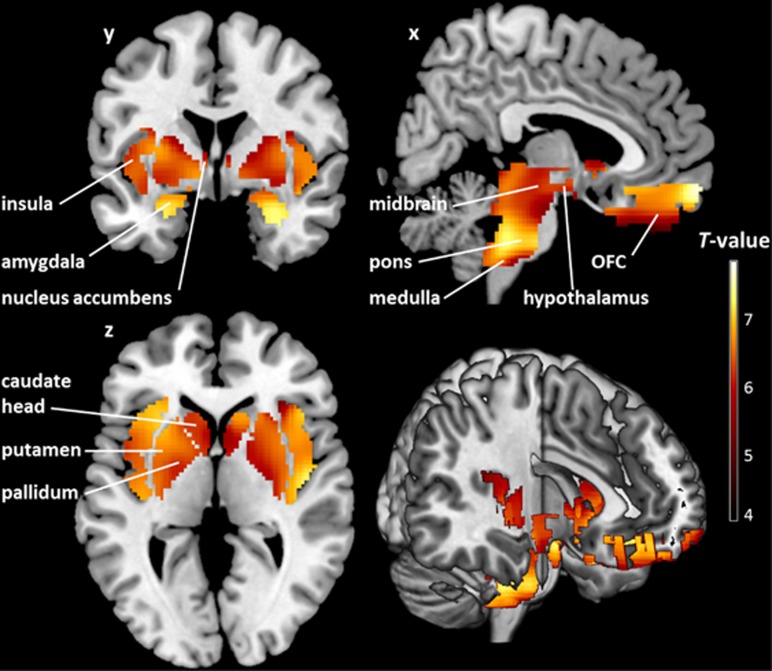
Brain regions where cannabinoid 1 receptor (CB_1_R) availability covaries negatively with log body mass index (BMI) in patients with food intake disorder (FID). *T* map of significant negative correlations between CB_1_R availability and log BMI in predefined homeostatic and reward-related regions of interest shown at a threshold of *P*_FWE-corrected_<0.05 (both on the voxel and cluster levels; *n*=54). The colored voxel-based statistical parametric mapping (SPM) results of the negative correlations in sagittal (*x*), coronal (*y*) and transverse (*z*) sections are overlaid on a normalized canonical image (ch2better-template) available in the MRICron software. The color bar expresses *T*-score levels.

SPM voxel-wise linear regression analysis at a voxel-level threshold of *P*_FWE-corrected_<0.05 revealed significant negative correlations between CB_1_R availability and log BMI in patients with FID in five clusters encompassing all homeostatic (hypothalamus, pons and medulla; all −1.06⩽*β*⩽−0.80, *P*⩽0.0001) and reward (midbrain, nucleus accumbens, caudate head, putamen, pallidum, orbitofrontal cortex, insula and amygdala; all −1.00⩽*β*⩽−0.89, *P*⩽0.0002) ROIs, accounting for a substantial amount of the variance (Supplementary Table 2). All clusters also survived the additional cluster-level threshold of *P*_FWE-corrected_<0.05 (Figure 1 and Table 3).

In keeping with these group results, additional exploratory analyses at an uncorrected significance threshold within each separate FID subgroup showed similar cluster patterns for AN, BN and FD, although not in OB. However, the latter might well be due to a ‘floor’ effect in CB_1_R availability within the OB group, as the variability in CB_1_R availability within this group (coefficient of variation, CV=0.11) was substantially smaller than in the other FID subgroups (AN, CV=0.19; BN, CV=0.21; FD, CV=0.18), leaving little variance to be explained by log BMI.

**Figure 2:**
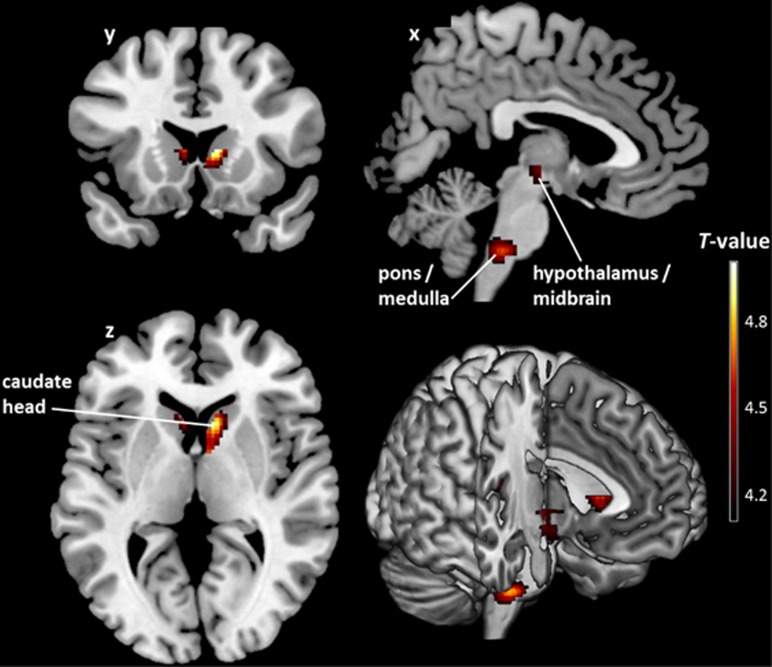
Brain regions where cannabinoid 1 receptor (CB_1_R) availability covaries negatively with log body mass index (BMI) in healthy controls. *T* map of significant negative correlations between CB_1_R availability and log BMI in predefined homeostatic and reward-related regions of interest shown at a threshold of *P*_FWE-corrected_<0.05 (both at the voxel- and cluster-level; *n*=26). The colored voxel-based statistical parametric mapping (SPM) results of the negative correlations in sagittal (*x*), coronal (*y*) and transverse (*z*) sections are overlaid on a normalized canonical image (ch2better-template) available in MRICron software. The color bar expresses *T*-score levels.

CB_1_R availability was also inversely correlated with log BMI in healthy subjects at a voxel-level threshold of *P*_FWE-corrected_<0.05, but only in a few regions predominantly involved in the homeostatic regulation of body weight and energy balance (hypothalamus, pons/medulla, caudate head and insula; all −0.63⩽*β*⩽−0.52, *P*<0.0004). All clusters were also significant at the additional cluster-level threshold of *P*_FWE-corrected_<0.05 (Figure 2, Table 3 and Supplementary Table 2).

Linear regression analysis between log BMI and eigenvariates extracted from a 5-mm sphere around the peak voxel of the clusters identified by the SPM analysis corroborated the voxel-wise regression analyses, as illustrated in Supplementary Figures 2 and 3. Important to mention is that we also obtained similar findings using BMI instead of log BMI for both FID patients and healthy controls (Supplementary Tables 3 and 4).

#### Conjunction and interaction analysis

We also wanted to identify brain areas within the mask where the association between CB_1_R availability and log BMI was either common to both groups or significantly different between FID and healthy subjects. Voxel-wise conjunction analysis at a voxel-level threshold of *P*_FWE-corrected_<0.05 revealed five clusters with shared negative correlations between the two groups. It has to be noted that these clusters were the same as those obtained in the control group (Figure 2 and Table 3), encompassing the hypothalamus (4.28⩽*T*⩽4.29), pons/medulla (*T*=4.74), caudate head (4.58⩽*T*⩽5.01) and insula (*T*=4.21). In contrast, the group-by-log BMI interaction analysis indicated that there were no clusters where the inverse association was significantly different between both groups, implying that the negative CB_1_R–BMI relationship in reward regions is more pronounced, rather than being categorically different between FID and controls. However, this might be due to the smaller sample size of the control group compared with the FID group, which lowers the power to detect such an interaction effect.

## Discussion

The psychobiological processes involved in (disordered) eating behavior and body weight regulation are complex and incompletely understood,^41^ but converging evidence points toward an important role of the neural circuits involved in the homeostatic and reward-related aspects of food intake, where the ECS has a vital role as neuromodulatory system.^42^ As a result, ECS dysfunction has become an auspicious pathophysiological mechanism and treatment target for several disorders of food intake, especially OB; however, the exact nature of this dysfunction remains unclear.

In the present study, we demonstrate for we believe the first time that CB_1_R availability in homeostatic and mesolimbic reward regions is inversely related to BMI in health and FID along the BMI continuum (that is, AN, BN, FD with weight loss, and OB). Specifically, our PET results show that lower CB_1_R levels in homeostatic brain areas such as the hypothalamus and brainstem are significantly associated with higher BMI in both healthy subjects and patients with FID. These findings indicate that variations in the endocannabinoid neurocircuitry in brain regions essential for energy balance regulation are inextricably linked to body weight, possibly reflecting a compensatory mechanism aimed at restoring energy homeostasis. However, in patients with FID along the BMI spectrum, additional negative correlations between CB_1_R availability and BMI were found throughout the mesolimbic reward system, including the midbrain, striatum and orbitofrontal cortex. This suggests that CB_1_R level deviations in brain areas implicated in encoding the incentive and hedonic value of food may have a role in the disordered hedonic eating behavior and body weight as observed in these patients.

Our data do not provide evidence for causality of the observed changes in CB_1_R levels. In our opinion, two possible interpretations are conceivable. First, these ECS changes may predispose subjects to aberrant body weight by interfering with the central regulation of appetite, food intake and energy balance. Alternatively, changes in CB_1_R availability might be a consequence of abnormal BMI and hence, indirectly, disturbed food intake. However, as there is currently no human evidence advocating one assumption over the other, future follow-up studies in subjects along the BMI spectrum should address the state- or trait-related nature of our findings. Furthermore, in both these explanations, the altered CB_1_R receptor availability may be a primary phenomenon or secondary to abnormal central endocannabinoid levels.

To date, only a limited amount of human data exist on the association between ECS function and BMI. Some peripheral components, including circulating plasma endocannabinoid levels,^43^ activity of the endocannabinoid-degrading enzyme fatty acid amide hydrolase in subcutaneous adipocytes,^44^ and perirenal visceral adipose tissue CB_1_R expression levels^45^ have been found to correlate positively with BMI in subjects ranging from normal weight to OB. In addition, a CB_1_R gene polymorphism was associated with lower BMI in healthy subjects with a wide BMI spread.^26^ However, most research has focused on ECS alterations within separate FID subgroups, especially AN and OB, which has led to the hypothesis of a (chronic) hypo- and hyperactivity of the (peripheral) ECS in, respectively, AN and OB conditions.^9, 21, 22, 23, 46, 47^ For example, upregulation of peripheral endocannabinoid signaling in overweight and OB individuals with and without binge-eating disorder has been demonstrated.^9, 21, 48^ Moreover, both animal and human studies have demonstrated the efficacy of CB_1_R antagonists/inverse agonists such as Rimonabant in reducing food intake and body weight in OB.^10, 11^ Preclinical studies have also shown increased hypothalamic endocannabinoid levels in diet-induced OB as well as several genetic models of OB.^19, 49^ It is suggested that this ECS overactivity in OB might originate from a high-fat diet and subsequent increased availability of polyunsaturated fatty-acid precursors for endocannabinoid biosynthesis. The hypothesis of ECS hypoactivity in anorectic conditions mostly originates from indirect evidence and animal work. Cannabinoid agonists such as dronabinol are used as therapeutic agents to treat AN and cachexia in cancer and AIDS patients.^50, 51^ Preclinical studies, addressing the effects of short-term starvation, have reported increased endocannabinoid levels in the limbic forebrain and hypothalamus of rats. However, in a context of prolonged starvation, reduced rather than increased brain endocannabinoid levels were observed throughout the entire mouse brain.^52, 53^ These apparent discrepancies can be interpreted as homeostatic endocannabinoid adaptations. In the short term, elevated endocannabinoid levels may be beneficial to trigger eating behavior, whereas in conditions of prolonged starvation (as in AN) this orexigenic mediator might be downregulated as an adaptive response to better cope with lack of food.^47, 53^

The presumed ECS hypoactivity in AN and hyperactivity in conditions of hyperphagia and OB is thought to be accompanied by, respectively, compensatory CB_1_R up- and downregulation. Using the same radiotracer, our group recently demonstrated increased CB_1_R binding in the AN and FD subgroups of this FID cohort^23, 31^ as well as the activity-based rodent model of AN.^24^ Conversely, CB_1_R downregulation in OB has been showed preclinically in forebrain and hindbrain regions.^20, 54^ These data clearly support our finding of an inverse association between CB_1_R availability and BMI in subjects across the BMI spectrum.

Although measurements of central endocannabinoid levels are impossible in humans *in vivo*, it is plausible that the negative CB_1_R–BMI correlation in our study represents the statistical embodiment of compensatory changes in CB_1_R availability, aimed at counteracting the above-mentioned aberrant endocannabinoid levels along the BMI continuum/FID spectrum. However, it has to be noted that deviations in CB_1_R levels could also occur independently from endocannabinoid content^55^ or follow changes of endocannabinoid tone in the same direction,^56^ as has been observed in AN^21^ and several other pathological conditions.^57^ Although differences in experimental methods can partly explain opposite findings within the same (food intake) disorder, this may well reflect the complexity of ECS regulation under pathological as well as physiological conditions.^13^ For example, it is uncertain whether peripheral endocannabinoid levels reflect the CNS status, as endocannabinoids are released on demand and rapidly metabolized in tissues.^23^ Measured (brain) tissue levels also do not necessarily reflect extracellular, and hence CB_1_R-active, content. However, whereas several explanations for the aberrant CB_1_R availability along the BMI spectrum are conceivable, we speculate that a compensatory mechanism (receptor desensitization and/or downregulation) is plausible from a large intracellular CB_1_R reserve.^58^ In support, other G-protein-coupled receptors (for example, serotonin 5-HT_1A_ and dopamine D_2_ receptors) are also inversely regulated by ligand availability.^59, 60^

Despite these interesting PET data, some caution is warranted when interpreting our results. Although we did find regional differences in the negative CB_1_R–BMI correlation between FID and CON, the group-by-BMI interaction analysis did not identify any areas where the negative correlation with BMI was significantly different between both groups. However, this could be because of the smaller sample size of the CON group compared with the FID, which lowers the power to detect such interaction effect. In addition, exploratory voxel-wise correlation analyses within the FID subgroups showed similar cluster patterns for AN, BN and FD but not OB. However, the latter might well be due to a ‘floor’ effect in CB_1_R availability within the OB group, as the variability in mSUV (reflecting CB_1_R availability) within this group was substantially smaller than in the other FID subgroups, leaving little variance to be explained by BMI. Moreover, confounding effects of weight differences on [^18^F]MK-9470 quantification are unlikely. Although the large differences in body weight between AN and OB may represent differences in [^18^F]MK-9470 distribution volume (*V*_T_), we have previously validated the use of mSUV in both patient groups and the activity-based rat model of AN, where full kinetic modeling showed a strong positive correlation (*R*^2^=0.9) of mSUV with V_T_.^24^ Moreover, the mSUV parameter is normalized by a reference weight to exclude possible confounding effects due to large weight differences between groups. In this way, the systematical underweight of AN patients would imply an underestimation of CB_1_R availability, whereas overweight would result in an overestimation. Hence, weight differences are not expected to explain the negative CB_1_R–BMI correlation observed in this study. Indeed, similar findings have recently been reported in both cannabis users and controls using a different CB_1_R tracer and quantification method, where a negative correlation between *V*_T_ and BMI was not driven by a peripheral confound.^61^ Finally, OB and part of the healthy control subjects were scanned using a different camera and, hence, also different acquisition protocols. However, our results remained unchanged when including camera/protocol as additional covariate of no interest in our analyses, thus excluding potential intercamera/protocol differences in CB_1_R assessment.

In conclusion, to the best of our knowledge, we demonstrate for the first time that CB_1_R availability in homeostatic brain regions is inversely related to BMI in both healthy subjects and patients with FID covering a wide BMI range (AN, BN, FD and OB). However, in FID, CB_1_R availability is also negatively correlated with BMI throughout the mesolimbic reward system. These results indicate that the cerebral homeostatic CB_1_R system is inextricably linked to BMI, with additional involvement of reward areas under conditions of disordered body weight. Thus, combined with (pre)clinical findings concerning peripheral ECS functioning, our results corroborate a key role for the ECS in body weight regulation and support the idea of pharmacological manipulation of the central ECS as a beneficial therapeutic approach for FID.

## Figures and Tables

**Table 1:** Demographic characteristics of patients with FID and healthy controls

*Characteristics (units)*	*FID*	*Healthy controls*	P*-value*
N	54	26	
AN	14		
BN	16		
FD	12		
OB	12		
			
Women (*n*)	53	25	> 0.99
Age (years)	29.3±12.6 (17.4–58.5)	34.6±15.3 (18.8–68.5)	0.10
BMI (kg/m^2^)	22.6±8.0 (12.5–40.6)	22.3±2.4 (18.5–26.6)	0.87
AN	15.5±1.3		
BN	21.8±2.5		
FD	18.4±2.6		
OB	36.1±3.4		
			
Injected activity of [^18^F]MK-9470 (MBq)	285.8±51.6 (128.7–387.2)	302.0±35.7 (167.3–340.8)	0.15

Abbreviations: AN, anorexia nervosa; BMI, body mass index; BN, bulimia nervosa; FD, functional dyspepsia with weight loss; FID, food intake disorder; OB, obesity.

Data are mean±s.d. Data range is represented between brackets.

**Table 2:** Peak voxels of brain areas where CB_1_R availability covaries negatively with log BMI in patients with FID

*Cluster*	*Cluster level*		*Voxel level*		*Peak voxel MNI coordinates*	*Anatomical localization*
	P_*FWE-corr*_	k_*E*_	P_*FWE-corr*_	T	x y z	
**1**	0.006	1118	<0.001	7.69	6 62 −14	Right medial orbitofrontal cortex
			<0.001	6.72	14 56 −18	Right superior orbitofrontal cortex
**2**	0.001	3637	<0.001	7.59	−30 12 −20	Left anterior insula
			<0.001	7.29	−30 −2 −28	Left amygdala
			<0.001	7.02	−28 10 6	Left putamen
			<0.001	6.81	−38 −20 2	Left posterior insula
			<0.001	6.76	−8 2 −6	Left globus pallidus
**3**	0.001	3607	<0.001	7.46	40 12 −16	Right anterior insula^a^
			<0.001	7.43	30 4 −28	Right amygdala
			<0.001	7.22	46 −10 2	Right posterior insula
			<0.001	7.16	8 18 −6	Right caudate head
			<0.001	6.60	20 20 −4	Right putamen
			<0.001	6.45	12 2 −6	Right globus pallidus
**4**	< 0.001	3827	<0.001	7.40	−14 −14 −16	Midbrain
			<0.001	7.31	6 −34 −36	Pons
			<0.001	7.16	6 −20 −38	Pons
			<0.001	6.92	10 −32 −10	Right hippocampus
			<0.001	6.88	−8 −42 −48	Medulla
			<0.001	6.26	−4 −4 −2	Hypothalamus
**5**	0.006	1150	<0.001	7.37	−2 58 −14	Left medial orbitofrontal cortex
			<0.001	6.75	−10 14 −22	Left superior orbitofrontal cortex
			<0.001	6.34	−42 48 −10	Left inferior orbitofrontal cortex

Abbreviations: BMI, body mass index; CB_1_R, cannabinoid 1 receptor; FID, food intake disorder; FWE-corr, family-wise error corrected for multiple comparisons; *T*, peak voxel *t*-statistic; *K*_E_, clus*t*er size extent; MNI, Montreal Neurological Institute.

The location and *t*-statistic of the local maxima of brain regions showing significant inverse correlations between log BMI and CB_1_R availability are presented (thresholded at *P*_FWE-corrected_<0.05, both at the voxel and cluster levels (*T*>4.2).

a Cluster also overlaps with nucleus accumbens.

**Table 3:** Peak voxels of brain areas where CB_1_R availability covaries negatively with log BMI in healthy controls

*Cluster*	*Cluster level*		*Voxel level*			*Peak voxel MNI coordinates*	*Anatomical localization*
	P_*FWE-corr*_	k_*E*_	P_*FWE-corr*_	T	P_*uncorr*_	x y z	
1	0.031	129	0.002	5.01	<0.001	10 16 6	Right caudate nucleus
2	0.028	168	0.006	4.74	<0.001	0 -32 -44	Pons/medulla
3	0.041	35	0.01	4.58	<0.001	-8 18 6	Left caudate nucleus
4	0.032	118	0.024	4.29	<0.001	-2 -8 -2	Hypothalamus^a^
5	0.043	24	0.029	4.21	<0.001	36 -18 16	Right insula

Abbreviations: BMI, body mass index; CB_1_R, cannabinoid 1 receptor; FWE-corr, family-wise error corrected for multiple comparisons; *T*, peak voxel *t*-statistic; *K*_E_, cluster size extent; MNI, Montreal Neurological Institute.

The location and *t*-statistic of the local maxima of brain regions showing significant inverse correlations between log BMI and CB_1_R availability are presented (thresholded at *P*_FWE-corrected_<0.05, both at the voxel and cluster levels (*T*>4.02)).

a Cluster also overlaps with the midbrain.
