# Supplementary material for: Association between cerebral cannabinoid 1 receptor availability and body mass index in patients with food intake disorders and healthy subjects: a [18F]MK-9470 PET study
Source: Transl Psychiatry. 2016 Jul 12;6(7):e853–. doi: 10.1038/tp.2016.118 (PMC5545708; doi:10.1038/tp.2016.118)
Supplement: Supplementary Information [file tp2016118x1.doc]

**SUPPLEMENTARY INFORMATION**

**Title:**

**Association between cerebral cannabinoid 1 receptor availability and body mass index in patients with food intake disorders and healthy subjects: a [^18^F]MK-9470 PET study**

**Authors:**

Jenny Ceccarini, Ph.D.^1*^ & Nathalie Weltens, M.Sc.^2*^, Huynh Giao Ly, Ph.D.^2^, Prof. Jan Tack, M.D., Ph.D.^2,3^, Prof. Lukas Van Oudenhove, M.D., Ph.D.^2,4,#^ and Prof. Koen Van Laere, M.D., Ph.D., D.Sc.^1,5,#^

^1^Division of Nuclear Medicine and Molecular Imaging, University Hospitals Leuven and Department of Imaging and Pathology, KU Leuven, 3000 Leuven, Belgium

^2^Translational Research Center for Gastrointestinal Disorders (TARGID), Department of Clinical and Experimental Medicine, KU Leuven, 3000 Leuven, Belgium

^3^Department of Gastroenterology, University Hospitals Leuven, 3000 Leuven, Belgium

^4^Liaison Psychiatry, University Psychiatric Center campus Gasthuisberg, University Hospitals Leuven, 3000 Leuven, Belgium

^5^LIND, Leuven Institute for Neurobiology and Disease, KU Leuven, 3000 Leuven, Belgium

*These authors contributed equally to this work as first authors

^#^These authors contributed equally to this work as senior authors

**Corresponding Author:**

Jenny Ceccarini, Ph.D.

Division of Nuclear Medicine and Molecular Imaging, Department of Imaging and Pathology, University Hospitals Leuven, KU Leuven, Herestraat 49, E901, 3000 Leuven, Belgium

E-mail: [jenny.ceccarini@uzleuven.be](mailto:jenny.ceccarini@uzleuven.be)

**SUBJECTS**

Restricting and binging-purging AN patients and purging BN patients diagnosed according to DSM-IV criteria were recruited during hospitalization in an inpatient university center for eating disorders. In order to assess psychological and clinical aspects related to eating disorders, AN and BN patients completed the Eating Disorder Evaluation Scale and the Eating Disorder Inventory[^1^](#_ENREF_1). FD patients were recruited from the (neuro)gastroenterology outpatient clinic of the University Hospitals Leuven. They had unintentional weight loss since the onset of their symptoms (on average mean±s.d. 11±2.1 kg) and were diagnosed with FD based on the Rome III criteria and complementary investigations[^2^](#_ENREF_2). Prior to inclusion, they were screened by means of self-report questionnaires and a neuropsychiatric interview (MINI)[^3^](#_ENREF_3). The severity of their dyspeptic symptoms was evaluated using the Dyspepsia Symptom Severity (DSS) scale[^4^](#_ENREF_4).

**SUPPLEMENTARY DATA**

**Supplementary Table 1: Predefined regions of interest**

| **Regions of Interest** | **Atlas** | **Lateralization** |
| --- | --- | --- |
| ***Homeostatic ROI*** |  | |
| Hypothalamus | TD Brodmann Areas + | Midline |
| Pons | TD Lobes | Midline |
| Medulla | TD Lobes | Midline |
| ***Reward ROI*** |  | |
| Midbrain | TD Lobes | Midline |
| Nucleus accumbens | IBASPM 71 | Left and right |
| Caudate head | TD Brodmann Areas + | Left and right |
| Putamen | IBASPM 116 | Left and right |
| Pallidum | AAL | Left and right |
| Insula | AAL | Left and right |
| OFC (BA11) | TD Brodmann Areas + | Midline |
| Amygdala | AAL | Left and right |

An anatomical mask of regions of interest was created using atlases within the WFU Pickatlas toolbox (SPM8). Abbreviations: ROI = region of interest; OFC = orbitofrontal cortex; BA = Brodmann Area; AAL = Anatomical Automatic Labeling; TD = Talairach Daemon; IBASPM 116 = Individual Brain Atlases using Statistical Parametric Mapping.

**Supplementary Table 2: Association between CB_1_R availability and log BMI in patients with food intake disorders and healthy controls**

|  | **Food intake disorders** | | | | | |  | | **Healthy controls** | | | | | | |  | |
| --- | --- | --- | --- | --- | --- | --- | --- | --- | --- | --- | --- | --- | --- | --- | --- | --- | --- |
| **Regions of interest** | **β** | **SE** | ***P-*value** | **standardized**  **β** | **R^2^** | |  | **β** | | | **SE** | ***P*-value** | **standardized**  **β** | | **R^2^** |  | |
| ***Homeostatic*** |  | | | | |  | | | |  | | | | | | |  |
| Hypothalamus | -0.40 | 0.12 | 0.0011 | -0.80 | 0.28 | |  | -1.09 | | | 0.28 | 0.0007 | -0.52 | 0.59 | | | |
| Pons | -0.47 | 0.10 | <0.0001 | -0.91 | 0.49 | |  | -0.98 | | | 0.24 | 0.0004 | -0.63 | 0.47 | | | |
| Medulla | -0.44 | 0.088 | <0.0001 | -1.06 | 0.40 | |  |  | | |  |  |  |  | | | |
| ***Hedonic*** |  | | | | |  | | | |  | | | | | | |  |
| Midbrain | -0.47 | 0.10 | <0.0001 | -0.97 | 0.38 | |  |  | | |  |  |  |  | | | |
| Caudate head | -0.59 | 0.14 | 0.0001 | -0.90 | 0.38 | |  | -1.39 | | | 0.33 | 0.0003 | -0.62 | 0.50 | | | |
| Putamen | -0.72 | 0.17 | 0.0001 | -0.94 | 0.33 | |  |  | | |  |  |  |  | | | |
| Pallidum | -0.57 | 0.14 | 0.0002 | -0.92 | 0.30 | |  |  | | |  |  |  |  | | | |
| Insula | -0.63 | 0.16 | 0.0002 | -0.89 | 0.36 | |  | -1.06 | | | 0.29 | 0.0015 | -0.49 | 0.58 | | | |
| OFC | -0.68 | 0.15 | <0.0001 | -1.00 | 0.38 | |  |  | | |  |  |  |  | | | |
| Amygdala | -0.60 | 0.13 | <0.0001 | -0.96 | 0.41 | |  |  | | |  |  |  |  | | | |

Results are parameter estimates of the linear regression analysis between the first eigenvariate of the peak voxel of all regions of interest surviving the SPM analysis and log BMI (see please Table 2 and Table 3 of the main article). Abbreviations: β = regression coefficient of log BMI; SE = standard error of the regression coefficient of log BMI; standardized β = standardized regression coefficient of log BMI; R^2^ = coefficient of determination.

**Supplementary Table 3: Comparison of linear regression analyses of CB_1_R availability and both log BMI and BMI in patients with food intake disorders**

|  | **Food intake disorders** | | | | | | | | | | | | | |
| --- | --- | --- | --- | --- | --- | --- | --- | --- | --- | --- | --- | --- | --- | --- |
|  | CB_1_R – log BMI | | | | | |  | | CB_1_R – BMI | | | | | |
| **Regions of interest** | **β** | **SE** | ***P-*value** | **standardized**  **β** | **R^2^** | |  | **β** | | | **SE** | ***P*-value** | **standardized**  **β** | **R^2^** |
| ***Homeostatic*** |  | | | | |  | | | |  | | | | |
| Hypothalamus | -0.40 | 0.12 | 0.0011 | -0.80 | 0.28 | |  | -0.023 | | | 0.0073 | 0.0023 | -0.91 | 0.31 |
| Pons | -0.47 | 0.10 | <0.0001 | -0.91 | 0.49 | |  | -0.019 | | | 0.005 | 0.0004 | -0.94 | 0.42 |
| Medulla | -0.44 | 0.088 | <0.0001 | -1.06 | 0.40 | |  | -0.020 | | | 0.0045 | < 0.0001 | -1.18 | 0.35 |
| ***Hedonic*** |  | | | | |  | | | |  | | | | |
| Midbrain | -0.47 | 0.10 | <0.0001 | -0.97 | 0.38 | |  | -0.020 | | | 0.0051 | 0.0003 | -1.04 | 0.35 |
| Caudate head | -0.59 | 0.14 | 0.0001 | -0.90 | 0.38 | |  | -0.026 | | | 0.0071 | 0.0006 | -0.97 | 0.34 |
| Putamen | -0.72 | 0.17 | 0.0001 | -0.94 | 0.33 | |  | -0.034 | | | 0.0094 | 0.0006 | -1.01 | 0.29 |
| Pallidum | -0.57 | 0.14 | 0.0002 | -0.92 | 0.30 | |  | -0.025 | | | 0.0073 | 0.0011 | -0.97 | 0.28 |
| Insula | -0.63 | 0.16 | 0.0002 | -0.89 | 0.36 | |  | -0.028 | | | 0.0078 | 0.0008 | -0.96 | 0.32 |
| OFC | -0.68 | 0.15 | <0.0001 | -1.00 | 0.38 | |  | -0.030 | | | 0.0074 | 0.0002 | -1.10 | 0.33 |
| Amygdala | -0.60 | 0.13 | <0.0001 | -0.96 | 0.41 | |  | -0.026 | | | 0.0066 | 0.0002 | -1.04 | 0.37 |

Values of CB_1_R availability were obtained by extracting the first eigenvariate from a 5 mm sphere around the peak voxel of the clusters identified by the SPM analysis. Abbreviations: β = regression coefficient; SE = standard error of the regression coefficient; standardized β = standardized regression coefficient; R^2^ = coefficient of determination.

**Supplementary Table 4: Comparison of linear regression analyses of CB_1_R availability and both log BMI and BMI in healthy controls**

|  | **Healthy controls** | | | | | | | | | | | | | | |
| --- | --- | --- | --- | --- | --- | --- | --- | --- | --- | --- | --- | --- | --- | --- | --- |
|  | CB_1_R – log BMI | | | | | |  | CB_1_R – BMI | | | | | | | |
| **Regions of interest** | **β** | **SE** | ***P-*value** | **standardized**  **β** | **R^2^** | |  | **β** | | **SE** | | ***P*-value** | | **standardized**  **β** | **R^2^** |
| ***Homeostatic*** |  | | | | |  | |  | | | | | | | |
| Hypothalamus | -1.09 | 0.28 | 0.0007 | -0.52 | 0.59 | |  | -0.051 | 0.012 | | 0.0004 | | -0.55 | | 0.60 |
| Pons | -0.98 | 0.24 | 0.0004 | -0.63 | 0.47 | |  | -0.045 | 0.011 | | 0.0004 | | -0.63 | | 0.46 |
| Medulla |  |
| ***Hedonic*** |  | | | | |  | |  | | | | | | | |
| Caudate head | -1.39 | 0.33 | 0.0003 | -0.62 | 0.50 | |  | -0.063 | 0.015 | | 0.0004 | | -0.62 | | 0.50 |
| Insula | -1.06 | 0.29 | 0.0015 | -0.49 | 0.58 | |  |  |  | |  | |  | |  |

Values of CB_1_R availability were obtained by extracting the first eigenvariate from a 5 mm sphere around the peak voxel of the clusters identified by the SPM analysis. Abbreviations: β = regression coefficient; SE = standard error of the regression coefficient; standardized β = standardized regression coefficient; R^2^ = coefficient of determination.

**Supplementary Figure 1: Relationship between mSUV and FUR values for patients with food intake disorders and healthy controls**

**
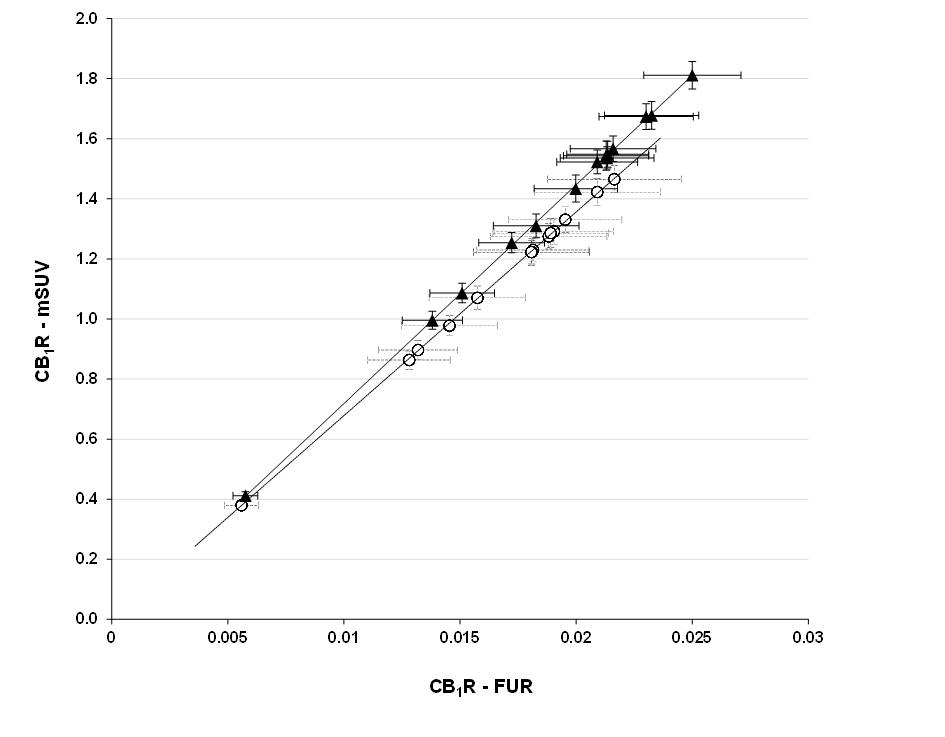
**

The relationship between mSUV (modified standard uptake value) and FUR (fractional uptake ratio) values for cortical and subcortical grey matter regions of interest in a subgroup of control subjects (*n* = 10; *R* = 0.99) versus patients with food intake disorders (*n* = 10; *R* = 0.99) is presented. The difference between the linear slopes is 6.6 %. Regions included were cerebellum, pons, white matter, thalamus, caudate nucleus, putamen, midbrain, anterior and posterior cingulate cortex, insula, frontal cortex, temporal cortex, parietal cortex, occipital cortex. Full triangles = food intake disorder patients, empty circles = healthy controls. Error bars indicate standard error of the mean.

**Supplementary Figure 2: Illustration of the peak inverse correlation between CB_1_R availability and log BMI in the hypothalamus and caudate head of patients with food intake disorders**

**
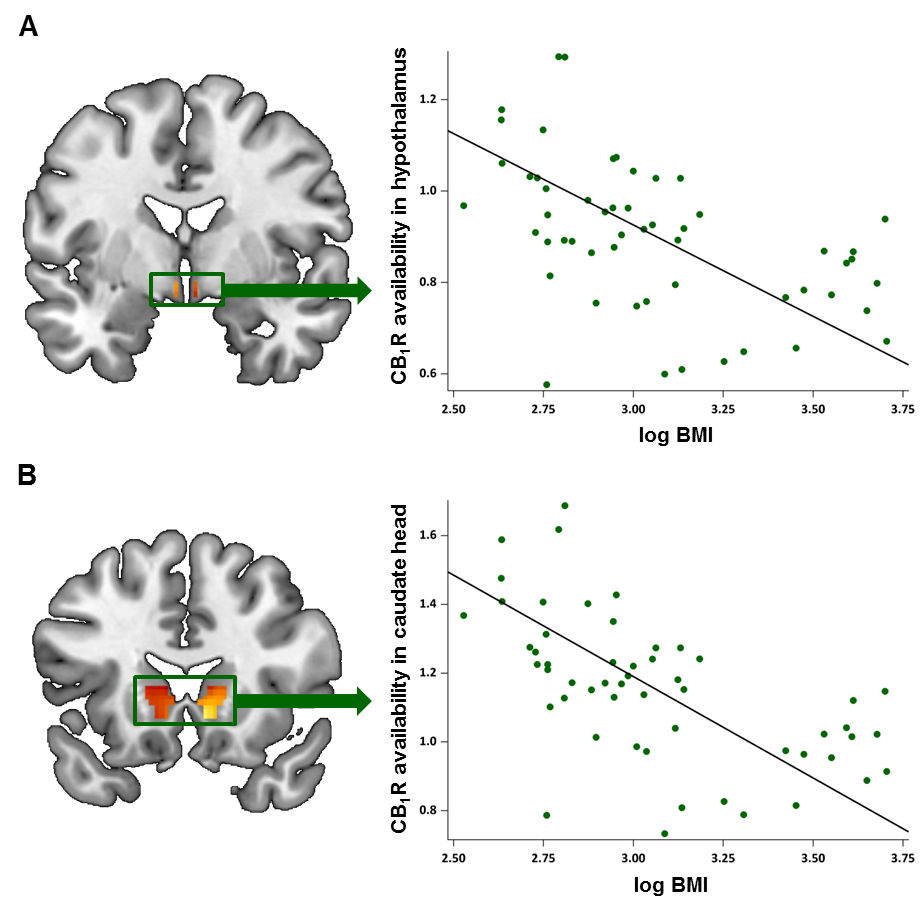
**

Statistical parametric maps superimposed on a normalized canonical image (ch2better template) using MRICron software (**left**) and correlation scatterplots representing the inverse association between log BMI and CB_1_R availability at the peak voxel (**right**) of (**A**) the hypothalamus (β = -0.40 ± 0.12; *P* = 0.0011) and (**B**) caudate head (β = -0.59 ± 0.14; *P* = 0.0001) in patients with food intake disorders. Values of CB_1_R availability were obtained by extracting the first eigenvariate from a 5 mm sphere around the peak voxel of the hypothalamus and caudate head (SPM8). Solid lines represent linear regression curves.

**Supplementary Figure 3: Illustration of the peak inverse correlation between CB_1_R availability and log BMI in the hypothalamus and caudate head of healthy controls**

**
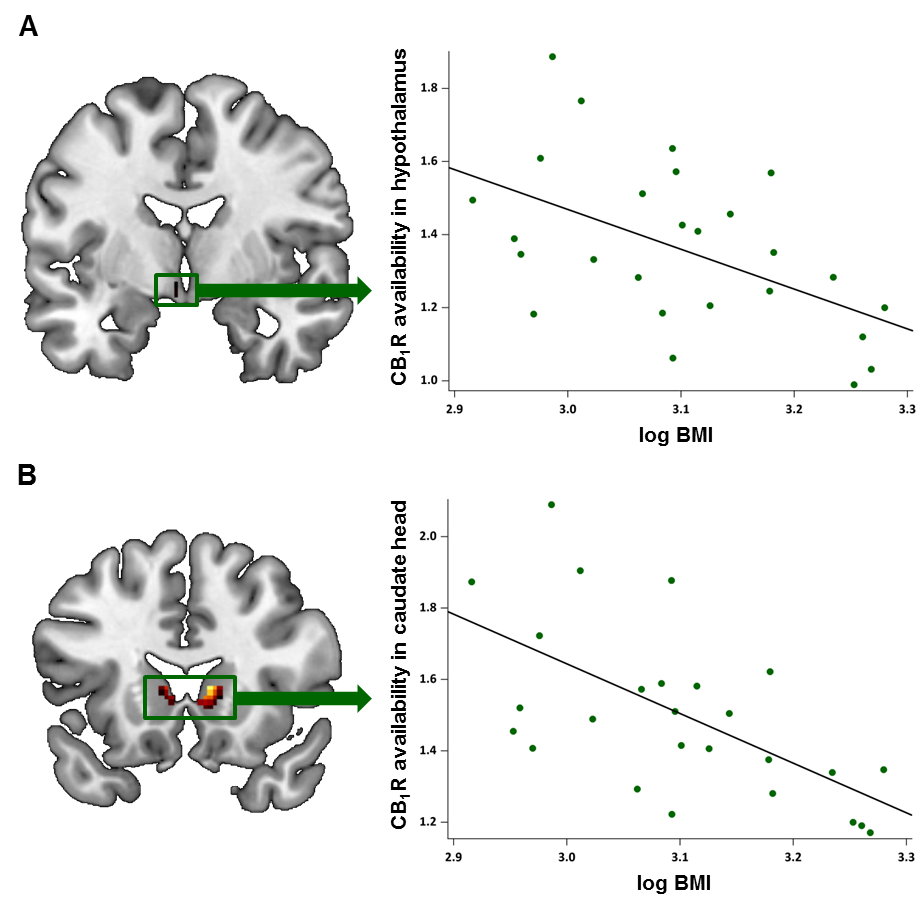
**

Statistical parametric maps superimposed on a normalized canonical image (ch2better template) using MRICron software (**left**) and correlation scatterplots representing the inverse correlation between log BMI and CB_1_R availability at the peak voxel (**right**) of (**A**) the hypothalamus (β = -1.09 ± 0.28; *P* = 0.0007) and (**B**) caudate head (β = -1.37 ± 0.33; *P* = 0.0003) in healthy controls. Values of CB_1_R availability were obtained by extracting the first eigenvariate from a 5 mm sphere around the peak voxel of the hypothalamus and caudate head (SPM8). Solid lines represent linear regression curves.

**REFERENCES**

1. Garner DM, Olmstead MP, Polivy J. Development and validation of a multidimensional eating disorder inventory for anorexia nervosa and bulimia. *International Journal of Eating Disorders* 1983; **2**(2)**:** 15-34.

2. Tack J, Talley NJ. Functional dyspepsia--symptoms, definitions and validity of the Rome III criteria. *Nature reviews Gastroenterology & hepatology* 2013; **10**(3)**:** 134-141.

3. van Vliet IM, de Beurs E. [The MINI-International Neuropsychiatric Interview. A brief structured diagnostic psychiatric interview for DSM-IV en ICD-10 psychiatric disorders]. *Tijdschrift voor psychiatrie* 2007; **49**(6)**:** 393-397.

4. Cuomo R, Sarnelli G, Grasso R, Bruzzese D, Pumpo R, Salomone M *et al.* Functional dyspepsia symptoms, gastric emptying and satiety provocative test: analysis of relationships. *Scandinavian journal of gastroenterology* 2001; **36**(10)**:** 1030-1036.
